# Highly Efficient Electrochemical Production of Hydrogen Peroxide Using the GDE Technology

**DOI:** 10.1021/acs.iecr.2c01669

**Published:** 2022-07-13

**Authors:** Paulo Jorge Marques Cordeiro-Junior, Justo Lobato Bajo, Marcos Roberto de Vasconcelos Lanza, Manuel Andrés Rodrigo Rodrigo

**Affiliations:** † São Carlos Institute of Chemistry, University of São Paulo (USP), Trabalhador São-carlense Street 400, 13566-590 São Carlos, SP, Brazil; ‡ Department of Chemical Engineering, Universidad de Castilla-La Mancha, Campus Universitario s/n, 13071 Ciudad Real, Spain

## Abstract

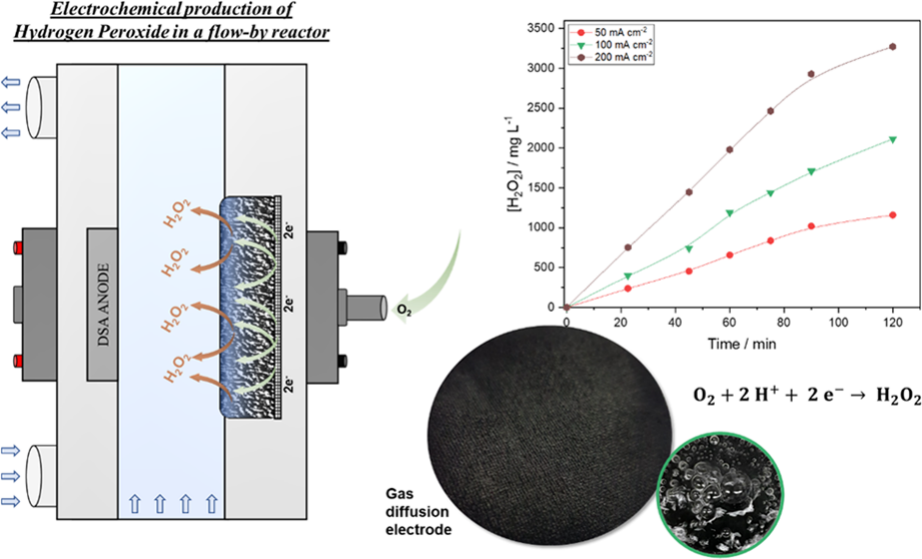

This work examines the role of oxygen supply in the improvement of the hydrogen peroxide (H_2_O_2_) electrochemical production efficiency and the generation of high H_2_O_2_ concentrations in electrochemical processes operated in a discontinuous mode. To conduct this study, a highly efficient Printex L6 carbon-based gas diffusion electrode (GDE) as a cathode was employed for the electrogeneration of H_2_O_2_ in a flow-by reactor and evaluated the effects of lowering the operation temperature (to increase solubility) and increasing the air supply in the system on H_2_O_2_ electrogeneration. The results obtained in this study show that unlike what is expected in flow-through reactors, the efficiency in the H_2_O_2_ production is not affected by the solubility of oxygen when GDE is employed in the electrochemical process (using the flow-by reactor); i.e., the efficiency of H_2_O_2_ production is not significantly dependent on O_2_ solubility, temperature, and pressure. The application of the proposed PL6C-based GDE led to the generation of accumulated H_2_O_2_ of over 3 g L^–1^ at a high current density. It should be noted, however, that the application of the electrocatalyst at lower current densities resulted in higher energy efficiency in terms of H_2_O_2_ production. Precisely, a specific production of H_2_O_2_ as high as 131 g kWh^–1^ was obtained at 25 mA cm^–2^; the energy efficiency (in terms of H_2_O_2_ production) values obtained in this study based on the application of the proposed GDE in a flow-by reactor at low current densities were found to be within the range of values recorded for H_2_O_2_ production techniques that employ flow-through reactors.

## 1 Introduction

The past few decades have seen a dramatic increase in the search for new technologies that are capable of producing chemical oxidants at substantial concentrations and in a highly efficient manner. Hydrogen peroxide (H_2_O_2_) is a highly efficient, eco-friendly chemical oxidant, which has a wide range of applications in different sectors.^1^ H_2_O_2_ has a high reduction potential (*E*^0^ = 1.77 vs standard hydrogen electrode, SHE) and produces nontoxic water when applied; as a result, this oxidant is widely applied in several industrial processes, including the synthesis of chemical products, paper bleaching, and wastewater treatment.^2−6^ As part of the efforts to combat the SARS-CoV-2 virus, H_2_O_2_ was widely employed as a reagent in the formulation of decontamination and disinfection products and for the cleaning of contaminated respiratory masks for reutilization due to its antimicrobial properties.^3^

The range of application of H_2_O_2_ has progressively increased in recent years, and the annual consumption of this oxidant is estimated to increase to 6 million tons in 2027.^2,7−9^ Most of the current production of H_2_O_2_ occurs through the anthraquinone process. To meet the increasing demand for H_2_O_2_, alternative efficient techniques for the production of the oxidant are currently being studied, and one of the techniques that have been found to be highly promising is the electrochemical production of H_2_O_2_ via oxygen reduction reactions (ORR)—see eq 1.^2,4,9−11^ The ORR technique employs oxygen as the raw material in the electrochemical process. Over the past few years, there has been a huge interest among researchers in the use of ORR via the 2-electron pathway for the electrogeneration of H_2_O_2_; this technique has become extremely popular because it is an energy-intensive multistep process, which has been proven to have the following advantages: high efficiency, good operational safety, and low environmental impact.^7,8^ There have been several reports in the literature regarding the mechanism of operation of the ORR process. As demonstrated in the literature, through the application of carbon-based cathode materials, O_2_ is easily reduced to H_2_O_2_ via the transfer of two electrons at a potential of 0.682 V vs SHE^4,11−13^
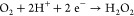
1Some studies reported in the literature have pointed out different ways to improve the production of H_2_O_2_ through ORR via the 2-electron pathway; some of these ways include the following:

(i) Improvement of the catalytic properties of the cathode; this can be done by studying and developing new highly efficient cathode materials based on carbon black (CB) or through the addition/doping of organic or inorganic catalysts into carbonaceous materials;^4,11,14−18^
(ii) Improvement of oxygen supply in the electrochemical cell since the low solubility of oxygen in the cell causes the efficiency of the process to be controlled by diffusion. As reported in the literature, to help tackle this problem, some important progress has been made by
  (a). performing the electrochemical operation under high pressure and at low temperature so as to improve oxygen solubility.^1,6,19−21^
  (b). optimizing the shape/configuration of carbon-based cathodes by employing gas diffusion electrodes (GDEs) in flow-by electrochemical reactors instead of flow-through electrode reactors.^1,18,19,22^
  (c). the design of more efficient electrochemical reactors with enhanced turbulence.^1,18,19^

So far, when it comes to the development and application of techniques for the production of H_2_O_2_ through ORR, most of the effort has been devoted toward the development of new cathodic materials (which use lab-scale cells) or specific applications of H_2_O_2_ (such as cells for wastewater treatment).^19,22−24^ No substantial effort has been devoted toward investigating and developing new efficient techniques that are capable of producing H_2_O_2_ at high concentrations.^14,16^ Thus, the present work aims to develop and optimize the operational parameters of a new carbon-based gas diffusion electrode (GDE) applied in a flow-by electrochemical reactor with a view to obtaining high H_2_O_2_ concentrations and high production efficiency. The choice of the electrochemical reactor operating mode to be flow-by is since the use of GDE in flow-by reactors has advantages in relation to the flow-through reactors, as it minimizes the formation of bubbles on the electrode surface, which increases the ohmic drop and also reduces the possibility of the electrolyte salt precipitation inside the GDE, blocking its channel structure and deactivating it over time.^25^

## 2 Experimental Section

### 2.1 Chemicals

The following reagents were used to perform the experiments: sodium sulfate (PanReac AppliChem), sulfuric acid (Scharlab), 60% w/w poly(tetrafluoroethylene) dispersion (PTFE—Uniflon), and titanium(IV) oxysulfate solution (Sigma-Aldrich). The aqueous solutions were prepared using ultrapure water (Milli-Q system with resistivity > 18 MΩ cm). Printex L6 carbon (PL6C) was purchased from Evonik Ltd. (Brazil).

### 2.2 Electrochemical Reactor Setup

**Figure 1:**
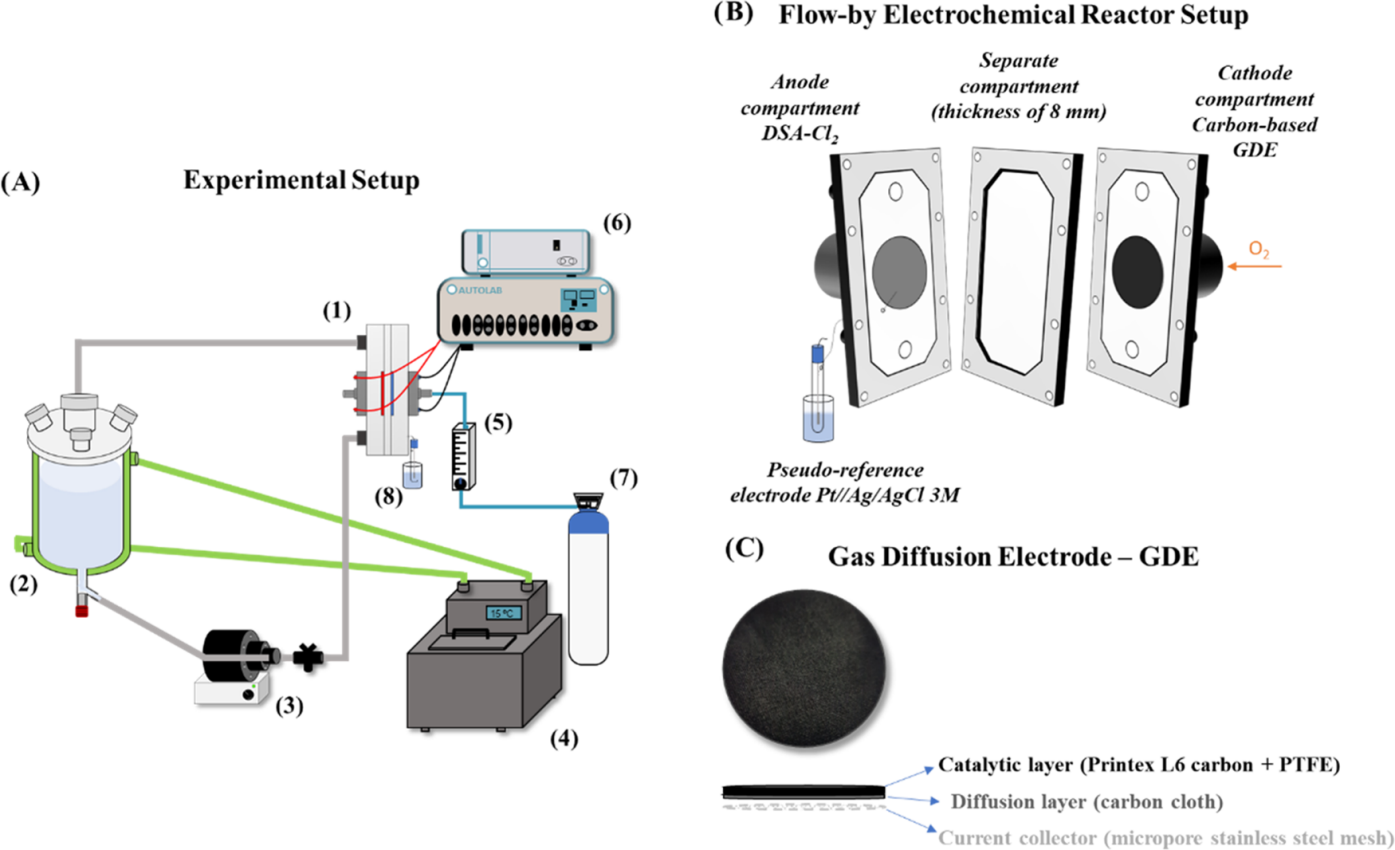
(A) Experimental setup: (1) electrochemical cell, (2) reservoir tank, (3) peristaltic pump, (4) thermostatic bath, (5) gas flowmeter, (6) power supply, (7) O_2_ gas cylinder, and (8) pseudo-reference electrode Pt//Ag/AgCl 3M. (B) Flow-by electrochemical reactor setup and (C) gas diffusion electrode composition.

As can be seen in Figure 1, the experimental system was set up using a flow-by electrochemical reactor with Printex L6 carbon/PTFE deposited on carbon cloth employed as the gas diffusion cathode and a dimensionally stable anode-chlor alkali (DSA-Cl_2_) used as the anode. The interelectrode gap was 8.0 mm, and both electrodes occupied a geometric area of 20.0 cm^2^. The electrolyte solution, 0.1 mol L^–1^ Na_2_SO_4_, pH 2.5, was fed to the electrochemical cell from the reservoir tank (with a capacity of 1.0 L) through a peristaltic pump operating at a flow rate of 50.0 L h^–1^ (under an electrolyte flow of 50 L h^–1^, the flow regime in the reactor is laminar, with a Reynolds number of ∼600 and an internal rate (*v*^0^) of ∼0.190 m s^–1^).^18^ A thermostatic bath coupled to the reservoir tank was used to control the temperature.

The flow-by electrochemical reactor used in this work operates under atmospheric pressure because the reactor is not hermetically closed due to the continuous entry of gas into the cathode compartment. O_2_ gas was continuously injected into the cathode compartment, and this was monitored with the aid of a gas flowmeter. An Autolab PGSTAT302N potentiostat coupled with a BOOSTER 10A was used as a power supply. A Pt//Ag/AgCl 3.0 M was employed as a pseudo-reference electrode and was coupled to the electrochemical cell, as described by Beati et al.^26^ The platinum wire and the Ag/AgCl reference electrode were added to an external chamber containing the same electrolyte as the electrolyte from the electrochemical reactor (0.1 mol L^–1^ of Na_2_SO_4_).

### 2.3 Preparation of Gas Diffusion Electrode

The carbon black (CB) material used for the conduct of the experiments was Printex L6 carbon (PL6C)—acquired from Degussa Brazil. Before manufacturing the GDE, the catalytic material—Printex L6 carbon is heat-treated at 120 °C for 24 h to remove water residues and organic interferences.^15^ After that, the carbon black material was mixed with 20 or 40% (w/w) of PTFE dispersion in 400 mL ultrapure water for 2 h until the mixture was completely homogenized. The catalytic mass was then filtered to remove excess water. Ten grams of the wet catalytic mass was deposited and spread over the carbon cloth (geometric area of 126 cm^2^). The electrode was dried at 120 °C for 15 min and was subsequently treated through the application of a pressure of 5 tons and a temperature of 290 °C for 2 h. The electrode was then cut into a circular shape of 20 cm^2^.

### 2.4 Electrochemical Study

The following experimental parameters were investigated in this study: (i) temperature (25, 15, and 5 °C); (ii) O_2_ flow applied to the cathode (10, 25, 50, 100, 200, and 300 mL min^–1^ or 0.5, 1.25, 2.5, 5, 10, and 15 cm min^–1^, respectively); (iii) PTFE (%) loading in the cathode composition (20 and 40%); and current density.

For the experimental tests (i) and (ii), electrolysis was performed by applying a constant current density of 50 mA cm^–2^ for a period of 60 and 120 min, respectively. The PTFE (%) loading was evaluated by electrolysis with different currents applied (25, 50, 75, 100, 125, 150, 175, and 200 mA cm^–2^).

### 2.5 Quantification of H_2_O_2_

Hydrogen peroxide was quantified (in mg L^–1^) using titanium(IV) oxysulfate solution as an indicator reagent, and the quantification analysis was performed by UV–vis spectroscopy (at λ = 408 nm) using an Agilent 300 Cary series UV–vis spectrophotometer. The method adopted for the quantification of H_2_O_2_ in this study was based on the technique proposed in previous studies reported in the literature.^1,6,19^

### 2.6 Service Lifetime Test

The lifetime of the electrode was evaluated by applying a current density of 200 mA cm^–2^ using Arbin Instruments (model FBTS—20 V). Cyclic voltammetry analysis was performed before and after the electrochemical durability tests in a potential window of 0.0 to −0.8 V and at a scan rate of 50 mV s^–1^ using Autolab PGSTAT302N equipment and Ag/AgCl 3M as a reference electrode.

### 2.7 Scanning Electron Microscopy (SEM) Images

The Printex L6 carbon-based GDE was morphologically characterized by scanning electron microscopy (SEM) using the HRSEM-Gemini-500 equipment. The images were taken with 40× magnification.

## 3 Results and Discussion

### 3.1 Effect of Temperature

One of the most efficient electrochemical experimental setups employed in the production of H_2_O_2_ via ORR through the 2-electron pathway involves the coupling of electrodes in a flow-through reactor operating at high pressure.^1,6,19^ The production of hydrogen peroxide is limited by the availability of oxygen on the cathode surface, and the solubility of this gas can be increased significantly when the system is operated under high pressure and at low temperature, as has been previously demonstrated.^1^ In addition, the low temperature applied in the process can help decrease the rate of H_2_O_2_ decomposition. By performing the electrolysis under these optimal conditions using a flow-by reactor in a discontinuously operated bench-scale plant, it was possible to obtain a maximum accumulated H_2_O_2_ concentration of approximately 400 mg L^–1^ at 0.9 Ah L^–1^ with a temperature of 11.5 °C and a pressure of 2 bar and a specific production of H_2_O_2_ of about 110 g kWh^–1^.^1^ The production of higher H_2_O_2_ concentration was not feasible in this discontinuous process because, under these conditions, there is an equilibrium between the rates of H_2_O_2_ production and decomposition, and from this point onwards, the process becomes unproductive. Thus, the only way to obtain higher H_2_O_2_ production efficiency is to change the operation mode from discontinuous to continuous mode,^1^ where the hydrogen peroxide removed is protected against self-decomposition.

**Figure 2:**
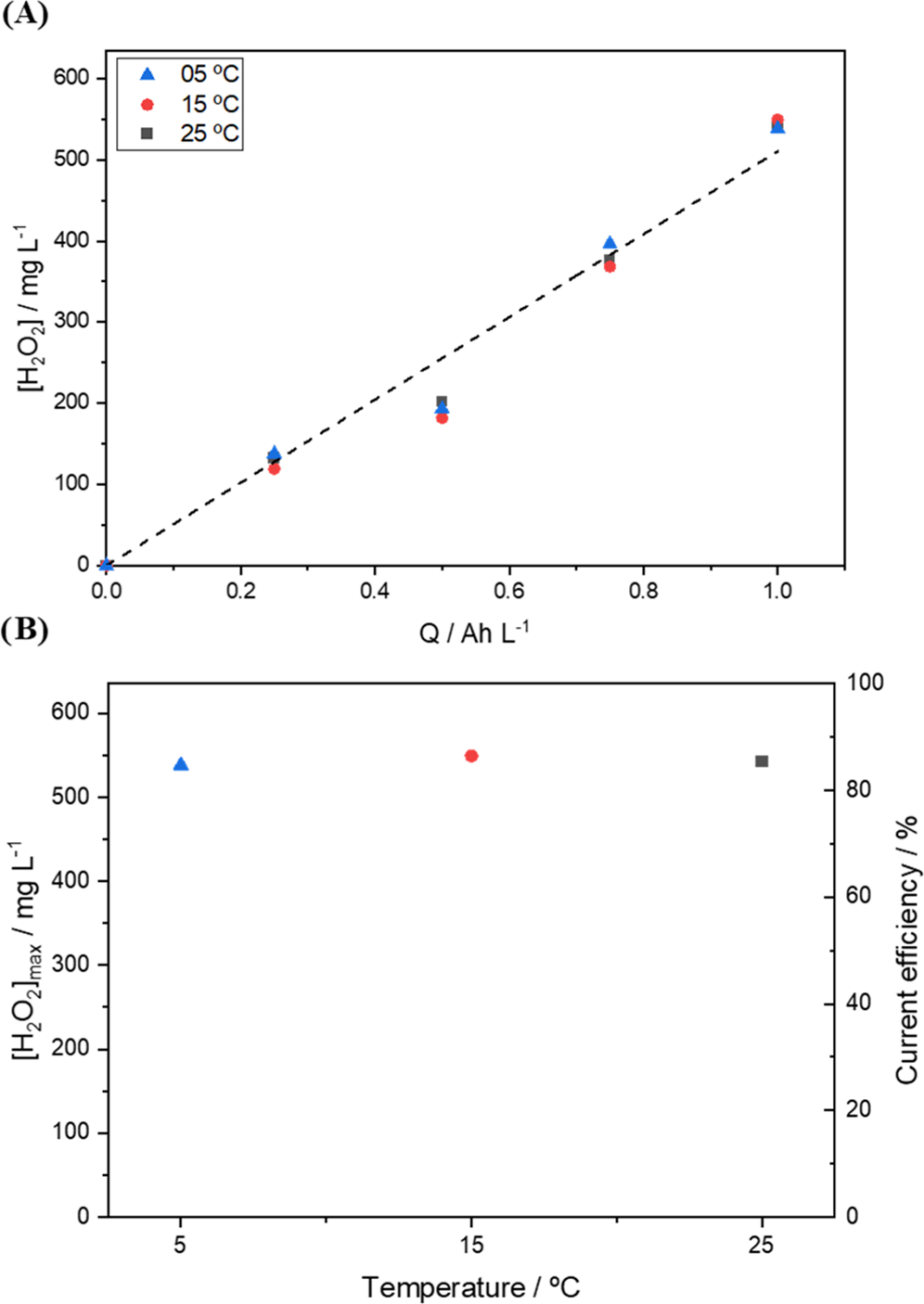
(A) Electrogeneration of H_2_O_2_ at different temperature levels (5.0, 15.0, and 25.0 °C); (B) maximum concentration of H_2_O_2_ produced in 60 min and current efficiency relative to the applied temperature for Printex L6 carbon employed at a current density of 50 mA cm^–2^ using 0.1 mol L^–1^ Na_2_SO_4_, at pH 2.5, as a supporting electrolyte. O_2_ flow rate employed: 2.5 cm min^–1^.

It should be noted that the physical mechanisms associated with the reduction of oxygen in GDE are very different from those that occur in electrodes made up of flow-through cells; furthermore, it is interesting to evaluate whether the decrease in temperature also exerts an influence over the physical mechanisms related to oxygen reduction in GDE. Thus, to evaluate the effects of temperature and O_2_ solubility in flow-by reactors using GDE, electrolysis experiments were carried out at 5, 15, and 25 °C, with a maximum O_2_ solubility of 14.8–11.2, 10.4, and 8.69 mg L^–1^, respectively. As can be seen in Figure 2A, unlike what is observed under the application of flow-through electrodes, the electrolyte temperature and O_2_ gas solubility do not play an influential role in the efficiency of GDE when applied in a flow-by electrochemical cell.

The electrolysis experiments carried out at 5, 15, and 25 °C, under atmospheric pressure and O_2_ flow at 50 mL min^–1^, yielded very similar concentrations of H_2_O_2_, with a mean value of 543 mg L^–1^ and a standard deviation of 5.6 mg L^–1^ at 1 Ah L^–1^. The average concentrations of H_2_O_2_ electrogenerated under the three temperature levels amounted a specific production of H_2_O_2_ of 93.5 g kWh^–1^—this is slightly lower than the values obtained for flat electrodes equipped in flow-through cells—the values reported for these electrodes ranged between 101 and 135 g kWh^–1^.^1,19^

Furthermore, as can be seen in eqs 2 and 3, the decomposition of H_2_O_2_ in the bulk solution or on the anode surface did not cause a decline in H_2_O_2_ concentration; this behavior was observed by Monteiro et. al in a flow-through cell.^1^ The main advantage of the flow-through electrochemical reactor is that the solution flow passes through the anode and cathode, which improves the oxidation or reduction rate, as well as the efficiency of the electrochemical process, because it improves the convection of material transfer in the electrode surface. According to Lu and Zhang,^27^ flow-through reactors have two advantages over the flow-by electrochemical reactors, which are (i) high mass transfer efficiency and (ii) electron-transfer efficiency. In the flow-by reactor, the mass transfer is highly limited by the fact that the electrodes are in parallel with the solution flow. Thus, the rate of decomposition of H_2_O_2_ is more pronounced in flow-through cells than in flow-by electrochemical cells; as such, the application of flow-through cells does not allow one to obtain higher electrogenerated H_2_O_2_ concentrations. It is worth emphasizing that the GDE works by increasing the efficiency of O_2_ mass transfer at the cathode, which improves this limitation of mass transfer in the flow-by electrochemical cell; however, the DSA-Cl_2_ anode, which is parallel to the GDE, is limited to the mass transfer
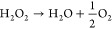
2
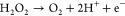
3It is noteworthy that the ORR process involving the production of H_2_O_2_ mostly occurs in the triple phase of the GDE since the O_2_ that is solubilized in the electrolyte does not practically interact with the electrode surface, and as such, it does not take part in the process. This phenomenon, observed in flow-through cells and which is characterized by the occurrence of higher H_2_O_2_ production efficiency at low temperature, would occur if H_2_O_2_ production was higher at 5 °C, once the solubility of O_2_ is higher at this temperature than at other temperature levels.

### 3.2 Effect of O_2_ Flow Rate

**Figure 3:**
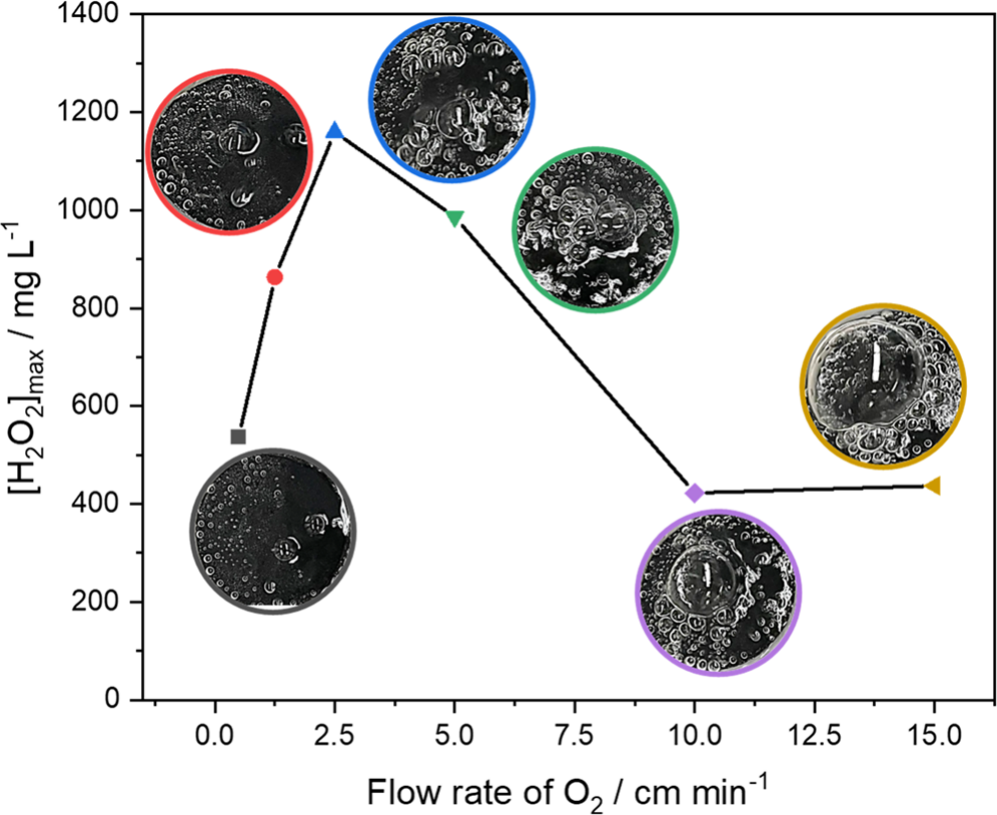
Maximum concentration of H_2_O_2_ produced in 120 min relative to the O_2_ flow rate. The experiments were performed at a current density of 50 mA cm^–2^ using 0.1 mol L^–1^ Na_2_SO_4_, at pH 2.5, as a supporting electrolyte.

As previously stated, the main limitation of the electrochemical production of H_2_O_2_ is the solubility of O_2_. High-pressured devices or accessories that promote the drag of bubbles such as the venturi mixer (improving the gas–liquid contact surface area) have been shown to enhance the efficiency of the electrochemical process by effectively supplying the O_2_ needed as a raw material.^6,19^ GDE is a suitable alternative device that helps to minimize the mass transfer constraint in the reactor, but the flow rate of O_2_ gas that passes through the cell may influence the performance of the electrochemical system in very different ways.^18,22^ In view of that, one needs to evaluate the O_2_ gas input so that there is no shortage or excess of the reagent, as this will impact the efficiency of H_2_O_2_ production. In the present study, the O_2_ gas injection flow into the cathode compartment was evaluated by varying the O_2_ reagent input from 0.5 to 15 cm min^–1^. The experimental conditions were kept at 15 °C. As can be seen in Figure 3, one can clearly observe that an increase in the gas flow resulted in an increase in the maximum concentration of H_2_O_2_ accumulated in the electrochemical device up to 1,159 ± 13.6 mg L^–1^ at 2.5 cm min^–1^. With regard to the O_2_ flow between 0.5 and 1.25 cm min^–1^, the amount of O_2_ recorded was lower; in other words, the oxygen did not interact with all of the ORR active sites available on the GDE and because of that the efficiency of H_2_O_2_ production and the accumulated concentration of H_2_O_2_ produced were found to be lower in the discontinuous process (536.4 and 863.1 mg L^–1^ for 0.5 and 1.25 cm min^–1^, respectively).

Looking at the images in the inset of Figure 3, one will observe the formation of large air pockets at conditions above 5 cm min^–1^; this is attributed to the excess gas that entered the cathode compartment, which increased in diameter as the injected O_2_ flow increased. At 15 cm min^–1^, the air pockets covered a large part of the electrode surface, reducing the three-phase contact area of the GDE; this resulted in a decrease in the amount of H_2_O_2_ accumulated by 510 mg L^–1^. This negative effect represented a 2.7-fold decrease in H_2_O_2_ production efficiency.

The nondependence of the GDE on O_2_ solubility and on its 3D-multichannel structure allows the electrode to provide an unlimited supply of oxygen at the electrode/electrolyte interface. The O_2_ that passes through the GDE can directly interact with the ORR active sites present in the Printex L6 carbon, and this promotes the generation of high concentrations of H_2_O_2_ in the system. In addition, the versatility of operation and easy installation of the GDE allow it to be operated in bench cells and in full-scale electrochemical reactors with volumes ranging from milliliters to hundreds of liters.

A point worth mentioning is that the O_2_ gas humidity does not play an important role in the ORR when using the GDE as a cathode. The catalytic layer of the GDE is almost totally hydrated due to the partial permeation of the electrolyte; in this way, when the O_2_ gas flow is passing through the channels of the GDE, the gas tends to increase its degree of humidity.^28^ Thus, regardless of the gas being prehumidified (relative humidity—RH of 50%) or dry (RH < 10%), a self-humidification occurs as the gas passes through multichannels of the GDE, as well as a decrease in proton resistance decreases due to water enrichment along the channel.^28^ Also, it is worth emphasizing that the oxygen reduction reaction must occur in an aqueous environment so that O_2_ is reduced to H_2_O_2_. Thus, it is essential that the catalytic layer of GDE is partially wet, and this parameter can be controlled by the PTFE content.

Xia et al. used gas diffusion electrodes based on carbon nanotubes with 40% PTFE loading to produce H_2_O_2_ in an undivided electrochemical cell with a volume of 0.16 L.^29^ The authors evaluated the effect of the O_2_ flow injected directly into the GDE—this was one of the parameters investigated in their study. Based on their results, the increase in O_2_ flow promoted an increase in O_2_ mass transfer within the GDE, and this in turn led to an increase in H_2_O_2_ production. However, when the O_2_ flow rate reached 280 mL min^–1^, there was a decline in the accumulated H_2_O_2_ concentration (compared to the O_2_ flow rate of 210 mL min^–1^).^29^ The excess of O_2_ flow led to the formation of bubbles that covered the surface of the electrode, and this led to a decrease in the production of H_2_O_2_. A similar outcome was noted in our present study. Xia et al. obtained the best H_2_O_2_ production efficiency at a flow rate of 210 mL min^–1^, where the accumulated H_2_O_2_ concentration was 1291 mg L^–1^ (with a current efficiency of 88.5% in 60 min of electrolysis).^29^ Remarkably, under the operating conditions employed by Xia et al.,^29^ the amount of O_2_ consumed was twice as high as the amount of O_2_ consumed in the present work even though our proposed system operated for an additional 1 h (1159 ± 13.6 mg L^–1^ in 120 min).

Another study that deserves being mentioned is that of Lima et al. where the authors employed a Printex L6 carbon-based GDE (similar to the electrocatalyst employed in our present study) to evaluate H_2_O_2_ production in an electrochemical cell. For comparison purposes, Lima et al. employed different amounts of carbon (8 g) and PTFE loading (40%) in their analysis (in the present study, 0.67 g carbon loading and 20% PTFE loading were employed).^24^ With the GDE exhibiting a relatively larger thickness due to the higher amount of carbon in its composition, the authors had to apply a pressure of 0.2 bar of O_2_ gas in the cathode compartment for the electrode to work in the best condition.^24^ Under these conditions, Lima et al. reported having obtained an accumulated H_2_O_2_ concentration of ∼750 mg L^–1^ after 120 min of electrolysis in an electrochemical cell. Interestingly, despite consuming a higher amount of reagent (O_2_), the amount of H_2_O_2_ concentration obtained in their study^24^ corresponds to only 65% of H_2_O_2_ concentration obtained from the application of the Printex L6-based GDE proposed in our present study. This shows that high amounts of carbon or high PTFE loadings are not required in the composition of the GDE since the ORR process, involving H_2_O_2_ production, occurs slightly below the electrode surface (on the catalytic layer of GDE), and thus the use of thinner electrodes can lead to satisfactory results.

### 3.3 Effect of PTFE (%) Loading and Current Density

The percentage content of PTFE employed in the GDE exerts an influential role on the hydrophobicity of the electrode; in other words, increasing the PTFE content in the GDE composition makes the electrode more hydrophobic and this inhibits the permeability of the aqueous solution through the electrode. With regard to the electrode proposed in the present study, the application of more than 40% PTFE in the GDE was found to render the device excessively hydrophobic, and this made the system behave like a conventional/flat electrode. On the other hand, the application of lower contents of PTFE resulted in higher permeability of the solution in the GDE (higher degree of wettability). With low contents of PTFE, the preliminary assays indicated that at values below 20%, the solution completely permeates the electrode according to the use of the GDE; this fact was observed by an electrolyte soaking in the cathode compartment when a GDE containing 10% PTFE was used. Thus, it was not possible to generate H_2_O_2_ electrosynthesis data for GDE containing values below 20%.

**Figure 4:**
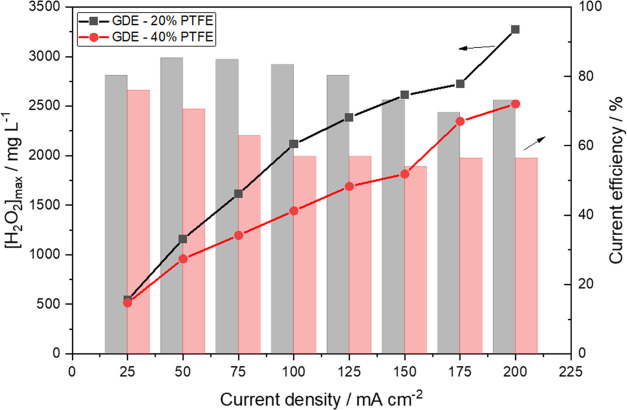
Maximum concentration of H_2_O_2_ obtained in 90 min of electrolysis based on the application of different current densities for each GDE. The supporting electrolyte employed: 0.1 mol L^–1^ Na_2_SO_4_, at pH 2.5.

It should be noted, however, that there is a minimum PTFE loading value that will prevent the solution from soaking through the electrode. Thus, it is essentially important to find an ideal PTFE loading that helps to prevent high hydrophobicity or high wettability of the electrode. The results obtained from the thorough analysis conducted in the present study helped obtain some useful insights in this regard. Based on our findings, the ideal PTFE loading should be between 20 and 40%; this is because below 20% PTFE loading, flooding occurs on the electrode, while there is high resistance to solution permeability when one applies a PTFE loading above 40% (see Figure 4).

A thorough investigation was carried out to evaluate H_2_O_2_ generation at different current densities using the GDE with PTFE loadings of 20 and 40% (see Figure 4 for the results obtained). One will observe that the accumulated H_2_O_2_ concentrations (obtained in 120 min of electrolysis) for the 20% PTFE–GDE were between 1.1- and 1.4-fold higher than those obtained for the 40% PTFE–GDE at all of the current densities evaluated. The 20% PTFE–GDE contains the equivalent of 80% of Printex L6 carbon by mass; this is 20% more than the amount of PL6C in the 40% PTFE–GDE.

The difference in the carbon content between the two electrodes (20% PTFE–GDE and 40% PTFE–GDE) resulted in an increase of almost 46% in current efficiency for the production of H_2_O_2_ at a current density of 100 mA cm^–2^; this effect can be attributed to a higher amount of ORR active sites available for O_2_ adsorption on the carbon surface. It is also worth emphasizing that lower contents of PTFE contain a higher amount of carbonaceous matrix (since the catalytic mass of GDE is composed of Printex L6 carbon and PTFE). The carbon matrix is responsible for promoting the electrochemical production of H_2_O_2_, and thus, the greater the content of carbonaceous material, the greater the amounts of ORR active site present in the GDE.

In previous works reported in the literature,^4^ it shows that Printex L6 carbon contains only carboxyl-type oxygenated functional groups (COOH) in its chemical composition (18.6% content—data referring to an analysis by X-ray photoelectron spectroscopy, XPS). As reported in the literature,^2,4^ the carboxyl group is the oxygenated functional group that has the greatest influence on H_2_O_2_ electrosynthesis, followed by the carbonyl functional group (C=O). The oxygenated functional group on the surface of the carbonaceous material is responsible for the displacement of electrons from its adjacent carbon, making it an excellent active site for the adsorption of the O_2_ molecule, and for tending to the formation of the OOH* intermediate, which is the only intermediate that favors the formation of H_2_O_2_ (the * symbolizes that the species is adsorbed at the active site).

Looking at Figure 4, one will observe that an increase in the current density resulted in an increase in the accumulated H_2_O_2_ concentrations obtained for both GDEs, but there was no ideal current density to work with. The accumulated H_2_O_2_ concentrations for 20% PTFE–GDE were 1614.3 ± 19.1, 2610.7 ± 40.6, and 3271.0 ± 47.3 mg L^–1^ at 75, 150, and 200 mA cm^–2^; for 40% PTFE–GDE were 1196.3 ± 14.3, 1443.1 ± 23.6, and 2523.9 ± 38.5 mg L^–1^ at 75, 150, and 200 mA cm^–2^, respectively.

**Figure 5:**
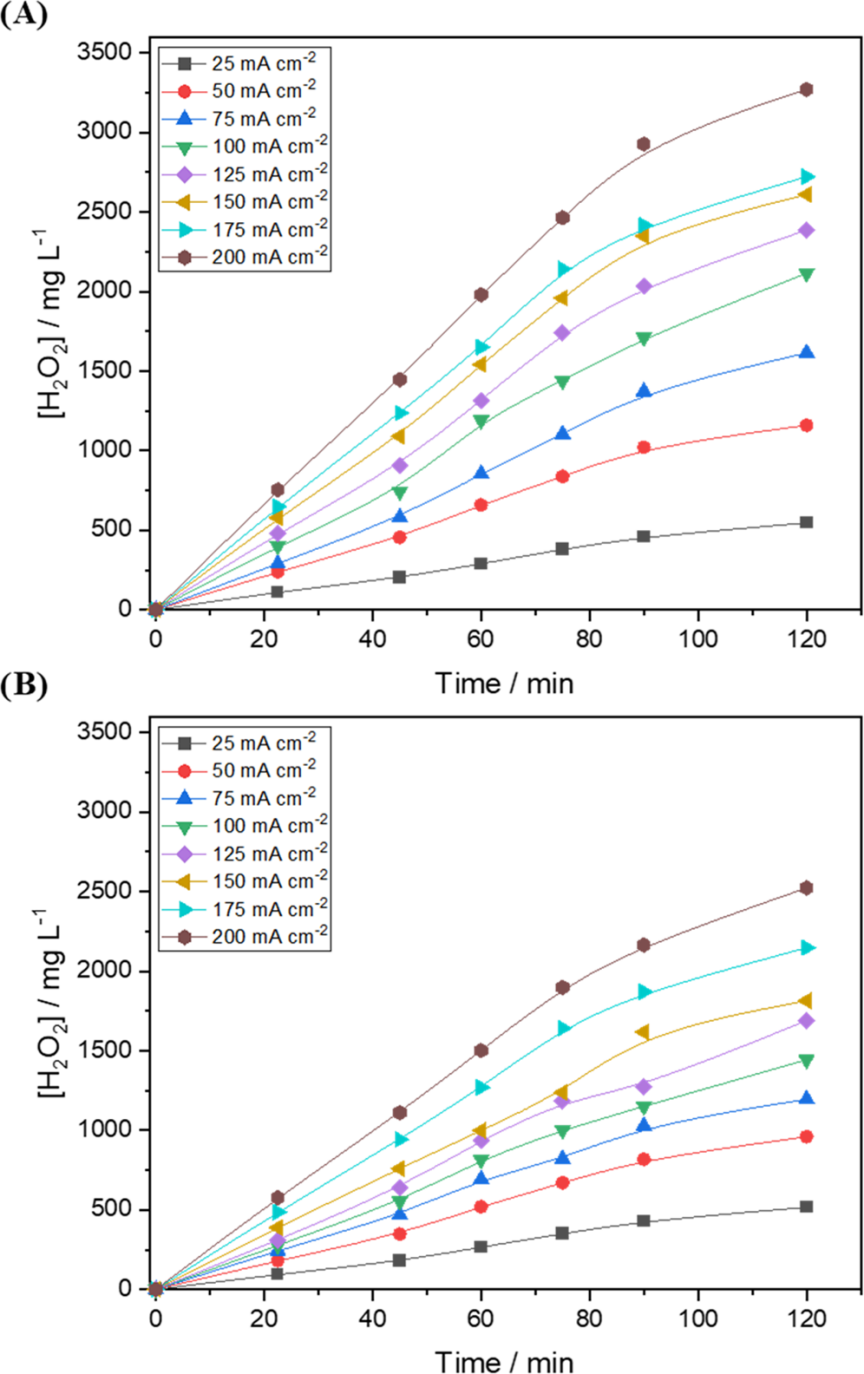
Amount of H_2_O_2_ electrogenerated (in mg L^–1^) for (A) Printex L6 carbon with 20% PTFE loading and (B) Printex L6 carbon with 40% PTFE loading at different current densities using 0.1 mol L^–1^ Na_2_SO_4_, at pH 2.5, as a supporting electrolyte. O_2_ flow rate: 2.5 cm min^–1^; electrolyte temperature: 15 °C.

Thus, for a better understanding of the electrochemical production of H_2_O_2_, one needs to observe Figure 5, which shows the relationship between the concentrations of H_2_O_2_ generated as a function of time for both GDEs. It can be noted that the application of current densities higher than 150 mA cm^–2^ led to a decrease in H_2_O_2_ concentration after 90 min of electrolysis due to the decomposition of H_2_O_2_ within the solution and on the anode surface (as discussed in eqs 2 and 3). The consumption of H_2_O_2_ by these parallel reactions causes a decline in the current efficiency; at very high current densities, the current efficiency may also decrease by favoring the ORR via 4-electron transfer on the cathode surface. The 20% PTFE–GDE exhibited a maximum current efficiency of 85.3% at a current density of 50 mA cm^–2^. From this value onwards (85.3%), the current efficiency only decreased until it reached 73% at 200 mA cm^–2^.

The same behavior (decline in current efficiency) was observed for the 40% PTFE–GDE; however, this electrode recorded a maximum current efficiency of 75.9% at a current density of 25 mA cm^–2^. Thus, one can conclude that for an operation aimed at obtaining a higher current efficiency, one needs to employ low current densities. However, when the aim is to obtain high H_2_O_2_ concentration in a short period of time, one will need to employ a high current density.

As can be seen in the first 60 min of the graph in Figure 5, the 20% PTFE–GDE recorded kinetic constant values that were 24, 47, 51, and 34% higher than those of the 40% PTFE–GDE at current densities of 50, 100, 150, and 200 mA cm^–2^, respectively. Both electrodes exhibited an apparent pseudo-order kinetic constant of zero (i.e., the generation of H_2_O_2_ is independent of the concentration of O_2_ and H^+^). The 20% PTFE–GDE showed H_2_O_2_ production rate values of 10.7, 15.5, 19.9, and 28.5 mg L^–1^ min^–1^ at current densities of 50, 100, 150, and 200 mA cm^–2^, respectively. Valim et al.^30^ reported a H_2_O_2_ production rate of 5.9 mg L^–1^ min^–1^ when operated at −1.0 V vs Ag/AgCl, whereas Carneiro et al.^15^ reported a slightly higher rate of 7.6 mg L^–1^ min^–1^ at the same conditions. In both works, the GDE was modified with metallic oxides, whose modification is to improve the selectivity and catalytic activity of the carbonaceous material. Moreira et al.^31^ reported surprising values of 38 mg L^–1^ min^–1^ when operated at 100 mA cm^–2^ using a Sudan-Red-modified Printex L6 carbon-based GDE.

**Figure 6:**
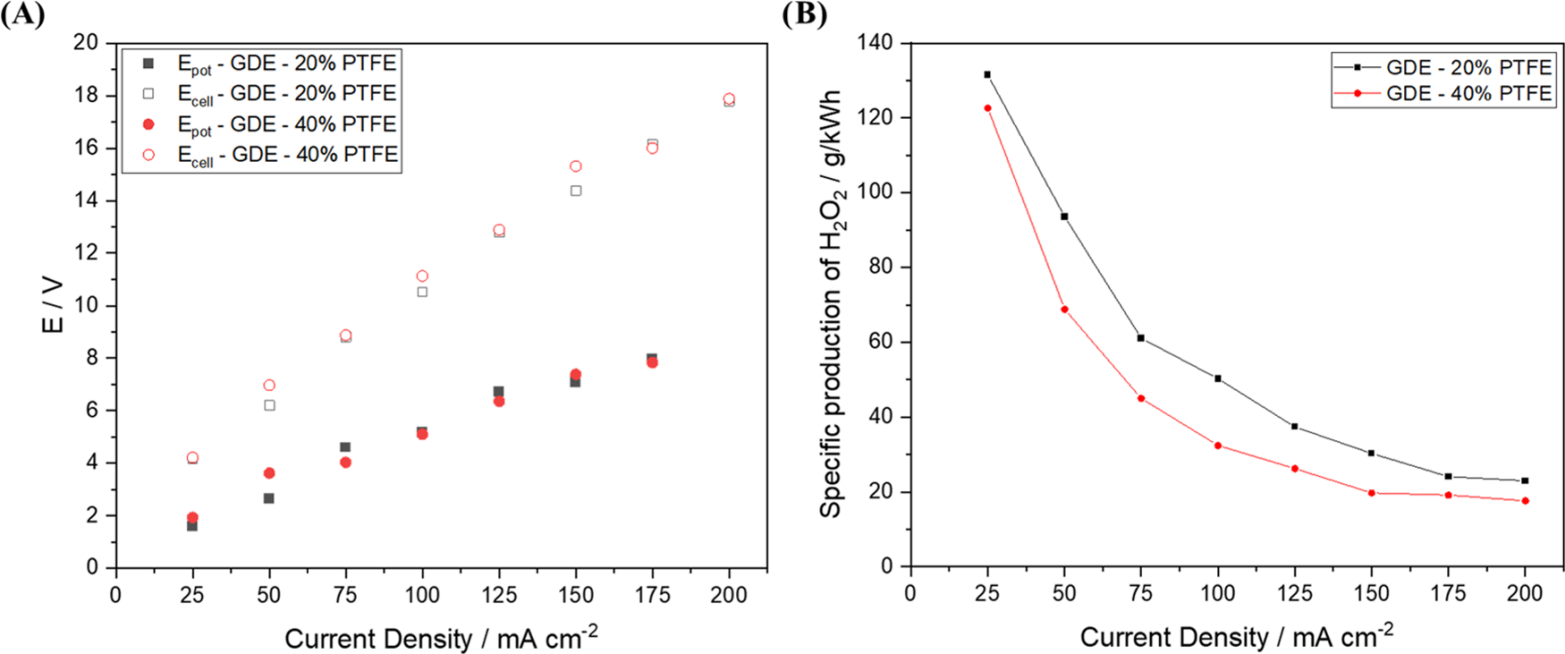
(A) Average cathode potential and cell potential values obtained for the electrodes investigated in 120 min of electrolysis at different current densities. (B) The specific production of H_2_O_2_ in g kWh^–1^ vs applied current density.

Finally, an interesting element to consider in our analysis of the efficiency of the electrochemical process is the cell potential (*E*_cell_) and the difference of potential between the cathode and pseudo-reference electrode Pt//Ag/AgCl (*E*_cat-ref_) values; this is because these potentials may indicate changes in the reactor setup or even in the electrode fabrication method. With that in mind, an analysis was conducted to evaluate whether the amount of carbon in the composition of the GDE can affect the cathode potential and cell potential values since it affects the conductivity of the electrode. Interestingly, both the 20% PTFE–GDE and 40% PTFE–GDE recorded very similar *E*_cat-ref_ and *E*_cell_ values, as seen in Figure 6A; this shows that both electrodes exhibit similar electrochemical behavior, despite the difference in carbon content.

As expected, an increase in the current density resulted in an increase in the potential values; also, higher *E*_cell_ values were recorded at higher current densities. This outcome suggests that, under these working conditions, the anode DSA-Cl_2_ plays an essential key role in the process, especially in the parallel reactions that lead to the decomposition of H_2_O_2_, as can be observed in eq 3 or in the formation of predator species for H_2_O_2_^21^ such as ozone, which plays a role in the decomposition of H_2_O_2_ once it is formed and disappears immediately afterward. It is worth noting that the use of more active anodes, such as BDD anode, can increase the intensity of parallel reactions and negatively influence the production of H_2_O_2_.^21^^21^

Based on the cell potential values, one can estimate the specific production of H_2_O_2_ in g kWh^–1^; this is an important and more realistic parameter that can help evaluate the applicability of the electrodes in real systems. The term specific production of H_2_O_2_ represents how much hydrogen peroxide is produced per power generated per time, which can be expressed in grams per kW per hour (g kWh^–1^). The specific production of H_2_O_2_ was calculated using eq 4, where C_H_2_O_2__ is the concentration of H_2_O_2_ (in mg L^–1^), *V* is the volume (in L), *E* is the cell potential (in V), *I* is the current (in A), and *t* is the time (in h)
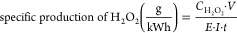
4Figure 6B shows the results obtained from the analysis of the specific production of H_2_O_2_ as a function of current density. One will observe that an increase in current density (*j*), which consequently results in an increase in the cell potential value, causes a decrease in the specific production of H_2_O_2_; in other words, smaller grams of H_2_O_2_ are produced per kWh at high current densities (the application of *j* > 100 mA cm^–2^ results in the specific production of H_2_O_2_ values less than 37 g kWh^–1^). On the other hand, at lower current density; i.e., 25 mA cm^–2^, an extremely high specific production of H_2_O_2_ values were recorded—the 20% PTFE–GDE and 40% PTFE–GDE recorded the specific production of H_2_O_2_ of 131.5 and 122.6 g kWh^–1^, respectively.

A comparison of the specific production of H_2_O_2_ values obtained for the proposed 20% PTFE–GDE and 40% PTFE–GDE with the values obtained for other gas diffusion electrodes reported in the literature pointed to the superior performance of the electrodes proposed in the present study. For illustration purposes, Lima et al.^24^ employed 40% PTFE–GDE at a current density of 50 mA cm^–2^, where they obtained a maximum H_2_O_2_ concentration of 750 mg L^–1^, with a specific production of H_2_O_2_ of ∼23.5 g kWh^–1^; this is roughly 2.9- and 4-fold smaller compared to the values obtained for the 40% PTFE–GDE and 20% PTFE–GDE proposed in our present work. Moreira et al.^31^ also employed 20% PTFE–GDE, where they obtained the specific production of H_2_O_2_ of ∼9.5 g kWh^–1^ at 100 mA cm^–2^; this is roughly 5 times lower than the value obtained in this work. Barros et al.^16^ employed an unmodified GDE for the electrochemical generation of H_2_O_2_ in a potentiostatic mode at −1.1 V (the cell current value should be approximately 150 mA cm^–2^), where they obtained a specific production of H_2_O_2_ of approximately 28 g kWh^–1^ and H_2_O_2_ accumulated a concentration of 6,400 mg L^–1^; a comparison of the conditions applied in their work with the GDE developed in our present work showed that our proposed GDE exhibited a slightly higher H_2_O_2_ production efficiency of 30 g kWh^–1^.

The efficiency in the production of H_2_O_2_ of the GDE developed in this work is because it is composed of a diffusion layer based on a carbon cloth and a catalytic layer based on Printex L6 carbon and PTFE. Some GDE reported in the literature (e.g., the work by Lima et al.,^24^ Moreira et al.,^31^ and Barros et al.^16^) employ a single layer that acts as both a diffusion and catalytic layer. In those case, up to the point at which the solution permeates the GDE, it is called the catalytic layer, while after this point, where the solution does not permeate, it is called the diffusion layer. The use of carbon cloth as the diffusion layer facilitates the diffusion of O_2_ gas to the catalytic layer, and therefore, it was possible to achieve a higher production of H_2_O_2_.

Flow-by electrochemical cells are characterized by higher cell voltage compared to pressurized nondivided microfluidic electrochemical cells. The main advantage of the pressurized nondivided microfluidic cells lies in the short separation distance between the cathode and the anode; it is this short distance between the cathode and the anode that allows these cells to have lower cell voltage and ohmic resistance compared to flow-by cells. It is worth noting that the lower the cell voltage, the less amount of specific production of H_2_O_2_. In previous studies conducted by Moratalla^19^ and Monteiro,^1^ the authors obtained the specific production of H_2_O_2_ values that ranged between 101 and 135 g kWh^–1^ at a current density of 5 mA cm^–2^; the values they obtained are slightly higher than those obtained in our present study at 25 mA cm^–2^. It should be noted, however, that the aforementioned studies [1, 20] employed different electrode technologies in the electrochemical process, which was more dependent on temperature and pressure.

### 3.4 Durability Test for GDE

**Figure 7:**
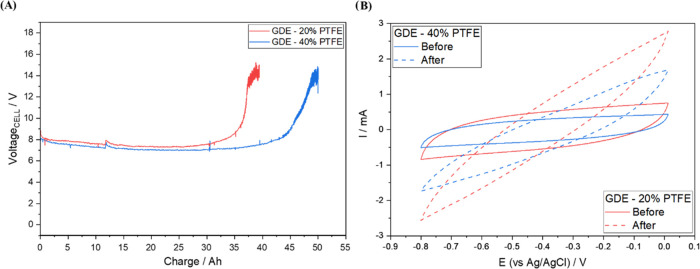
(A) Durability test for 20% PTFE–GDE (red line) and 40% PTFE–GDE (blue line) at a current density of 200 mA cm^–2^; (B) cyclic voltammetry analysis performed on the cathode potential (in the potential range of 0.0 to −0.8 V and at a scan rate of 50 mV s^–1^) before and after the durability test using O_2_-saturated 0.1 mol L^–1^ Na_2_SO_4_ (pH 2.5 adjusted with H_2_SO_4_) as an electrolyte solution.

The electrochemical resistance of the 20% PTFE–GDE and 40% PTFE–GDE was evaluated by applying a current density of 200 mA cm^–2^ and the operation time needed for the *E*_cell_ to increase exponentially. As can be seen in Figure 7A, the electrode containing 20% of PTFE loading maintained the *E*_cell_ constant for 36 Ah (this corresponds to 7.5 days), after which the voltage increased significantly. The electrode with 40% PTFE loading exhibited a longer lifetime, reaching an uninterrupted life span of 48 Ah (10 days). After the aforementioned lifetime, both GDEs exhibited a more resistive current profile, as can be seen in the cyclic voltammograms obtained before and after the durability test (see Figure 7B); this behavior can be attributed to the fact that the electrodes lost a significant part of the catalytic film (the catalytic mass containing Printex L6 carbon and PTFE) deposited under the carbon cloth. In addition, the GDEs were found to have been soaked by the electrolyte.

**Figure 8:**
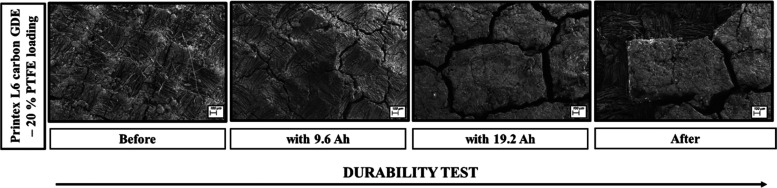
SEM images (with 40× magnification) obtained for 20% PTFE–GDE before and after the durability test.

Figure 8 shows the SEM images related to the removal of the catalytic film from the carbon cloth in the 20% PTFE–GDE. Before the durability test, the catalytic mass was deposited homogeneously and uniformly over the carbon cloth substrate (Figure 8, before). During the durability test, the catalytic film (Printex L6 carbon + PTFE) started to exhibit some cracks (like cracked soil). An increase was observed in the thickness of the cracks over time until parts of the catalytic film were removed from the carbon cloth substrate (Figure 8, after).

## 4 Conclusions

The present work reported the development and application of a new optimized carbon-based gas diffusion electrode supported on carbon cloth employed as a cathode in a flow-by electrochemical reactor for the electrochemical production of H_2_O_2_. The following conclusions can be drawn from the results obtained in this study:

- The use of the proposed PTFE–GDE (as cathode) in a flow-by reactor helped eliminate the dependence of the electrochemical process on temperature and O_2_ solubility in terms of the electrochemical production of H_2_O_2_; the application of the proposed system allowed O_2_ to pass through the 3D-multichannel structure of the GDE until it reached the triple phase where the ORR active sites were located, giving rise to the production of H_2_O_2_. The application of the following temperature levels, 5, 15, and 25 °C, resulted in the average H_2_O_2_ production of 8.51 mg L^–1^ min^–1^ and a specific production of H_2_O_2_ of 93.5 g kWh^–1^.
- An analysis of the mass transfer of O_2_ injected into the cathode compartment showed that the ideal O_2_ flow rate was 2.5 cm min^–1^; the application of this O_2_ flow rate in the electrochemical system led to the production of 1,159 ± 13.6 mg L^–1^ of H_2_O_2_ in 120 min of electrolysis. The application of an O_2_ flow rate below 2.5 cm min^–1^ caused a significant decrease in mass transfer, and this undermined the interaction of O_2_ with the active sites of the ORR; in these conditions, large air pockets were formed, which covered a great part of the electrode surface, reducing the H_2_O_2_ production efficiency.
- The hydrophobic characteristic of the GDE is determined by the PTFE loading. The findings of this study showed that the application of GDE composed of PTFE loading below 20% led to the soaking of the electrode, while the incorporation of PTFE loading above 40% into the GDE elevated the resistance of the electrode to partial permeability, impeding the smooth operation of the triple phase. The 20% PTFE–GDE sample produced a high accumulated H_2_O_2_ concentration, which was 1.4 times higher than the amount obtained for the 40% PTFE–GDE.
- The application of low current densities favored the current efficiency and H_2_O_2_ production efficiency; the application of the current density of 50 mA cm^–2^ resulted in current efficiency and specific production of H_2_O_2_ of 80.3% and 131 g kWh^–1^, respectively. It should be noted, however, that the application of a current density of 200 mA cm^–2^ in 120 min of electrolysis led to the production of an accumulated H_2_O_2_ concentration of nearly 3,270 ± 47.3 mg L^–1^ but with a low current efficiency of 64.5% and a specific production of H_2_O_2_ of 23 g kWh^–1^. This is attributed to the increase in parallel reaction rates associated with H_2_O_2_ decomposition as a result of an increase in applied current density.
- The durability/lifetime test conducted showed that the 40% PTFE–GDE recorded a lifetime of 48 Ah (which corresponds to 10 days of uninterrupted use) at 200 mA cm^–2^. The lifetime of the 40% PTFE–GDE was found to be 1.3 times higher than that of the 20% PTFE–GDE; in essence, this result shows that an increase of the PTFE loading in the GDE resulted in an increase in the electrode lifetime. Over time, the catalytic film showed surface cracks, which increased in thickness until the film was removed from the substrate.
